# A Bootstrap Method for Goodness of Fit and Model Selection with a Single Observed Network

**DOI:** 10.1038/s41598-019-53166-6

**Published:** 2019-11-13

**Authors:** Sixing Chen, Jukka-Pekka Onnela

**Affiliations:** Department of Biostatistics, Harvard T.H. Chan School of Public Health, 655 Huntington Ave, SPH2, 4th Floor, Boston, MA 02115 USA

**Keywords:** Computational models, Statistics, Computational science

## Abstract

Network models are applied in numerous domains where data arise from systems of interactions among pairs of actors. Both statistical and mechanistic network models are increasingly capable of capturing various dependencies among these actors. Yet, these dependencies pose statistical challenges for analyzing such data, especially when the data set comprises only a single observation of one network, often leading to intractable likelihoods regardless of the modeling paradigm and limiting the application of existing statistical methods for networks. We explore a subsampling bootstrap procedure to serve as the basis for goodness of fit and model selection with a single observed network that circumvents the intractability of such likelihoods. Our approach is based on flexible resampling distributions formed from the single observed network, allowing for more nuanced and higher dimensional comparisons than point estimates of quantities of interest. We include worked examples for model selection, with simulation, and assessment of goodness of fit, with duplication-divergence model fits for yeast (S.cerevisiae) protein-protein interaction data from the literature. The proposed approach produces a flexible resampling distribution that can be based on any network statistics of one’s choosing and can be employed for both statistical and mechanistic network models.

## Introduction

Networks are used to represent data from systems composed of interactions among pairs of actors (represented by nodes)^1–5^. Often in such systems, these interactions (represented by edges) can depend on the state of the rest of the system, such as other edges or attributes of nodes. One prominent example of this is triadic closure in social networks, where two people are more likely to be friends should they share a mutual friend^6^. While innovations in network models are increasing the capability to account for various dependencies in the data, this rich level of interconnectedness poses a problem for statistical methods for networks.

In typical statistical settings, the premise is that the data is composed of a collection of independent observations. Typical methods derive efficiency gains and consistency from a large number of samples due to this independence. However, in the network context where the structure of the network is of primary interest, the edges and their placement are the outcome of interest, but there are often multiple layers of dependence. Thus, the premise of independent observations is not met and most statistical methods are not applicable.

To better understand the limitations, we inspect two prominent paradigms of network models. First, statistical models are probabilistic models that specify the likelihood of observing any given network^7–9^. One example of these models is the family of exponential random graph models (ERGMs)^4^, which uses observable network configurations (such as triangles and *k*-stars) as the natural sufficient statistics. Although popular in practice, ERGMs can be difficult to fit and to sample from, and they may not scale well to large networks^10^. Estimation of ERGMs is done using maximum pseudolikelihood estimation (MPLE)^11^ or Markov chain Monte Carlo maximum likelihood estimation (MCMC-MLE)^12,13^. Pseudolikelihood methods for inference with ERGMs can lead to biased results due to the ignored dependence^14^, while inference for MCMC-MLE proceeds via simulation from estimated model^13^ and is thus entirely model based. Second, mechanistic models are composed of generative mechanisms that prescribe the growth and change of a network^15–20^. While they are easy to sample from, a mechanistic model allows for numerous paths that can be taken in the state space to produce any one observed network, making the likelihood (of all but the most trivial models) intractable. As a result, performing statistical procedures is difficult for such models and there is little existing work in the literature for doing so.

In situations where likelihood based methods are not available, one often resorts to resampling methods, such as bootstrap, jackknife, and permutation tests^21–23^. Although the different resampling methods operate differently, they all serve to create new data sets from a single observed data set that mimic the behavior of the original one to serve as a basis for statistical procedures. This is an attractive option for networks, especially if there is only a single observed network, such as the Internet or the World Wide Web. Having multiple resampled networks that resemble, in some ways, the original observed network allows one to bypass the problem of unwieldy or intractable likelihoods. Even in the best case of the likelihood having a simple functional form, the normalizing constants of ERGMs are generally unobtainable even for a network of modest size, since they require summing over a computationally infeasible number of possible network realizations. For example, in a network of *m* edges, one may need to consider all the possible *m*! orders of adding the edges since the mechanisms of growth may depend on the existing state of the network.

In this paper, we explore using a resampling procedure as a basis for statistical procedures for a single observed network. While there is some existing research on resampling methods in network settings, our approach is distinct in many ways. First, there are methods for assessing the goodness of fit for a fitted model^24,25^. These methods generally work by drawing network realizations from the fitted model, and then assessing fit by comparing the value of a set of network statistics for the observed network to the distribution of these statistics in the generated draws. This resampling scheme is akin to that of the parametric bootstrap. Note that this can be done for the point estimate of individual statistics or those of multiple statistics simultaneously, e.g., functionals of the degree distribution. However, the resamples in these methods are only representative of the *fitted model* and not necessarily of the *observed network*, and comparisons are made based only on point estimates. Second, there are methods for a setting where there are multiple independent networks observed for maximum pseudolikelihood estimation (MPLE)^26^. This is similar to the typical statistical setting with multiple independent observations and is not applicable to the setting with a single observed network. Third, there are resampling methods based on subgraphs of subsamples of nodes in the observed network^27–31^. Ohara *et al*.^27^, Bhattacharyya *et al*.^28^, Thompson *et al*.^30^, and Gel *et al*.^31^ are aimed at estimation and uncertainty quantification of network centrality, distribution of subgraphs, and functionals of the degree distribution, while Ali *et al*.^29^ is a subgraph-based method for network comparisons.

Our procedure makes use of the bootstrap subsampling scheme from Bhattacharyya *et al*.^28^. Importantly, our method addresses goodness of fit and model selection rather than estimation, and is based on the entire resampling distribution (rather than point estimates) of any set of statistics obtained from the sampled subgraphs. The flexible choice of network statistics allows an investigator to focus the criterion for model fit based on scientific interest. The full resampling distribution also contains more information than aggregated subgraph counts and point estimates for comparison with candidate models. The procedure also allows for natural uncertainty quantification regardless of the algorithm used for selecting the model, is agnostic to the modeling paradigm (statistical or mechanistic), and can accommodate any model one can sample from. The scaling of the procedure depends on the statistics chosen and the number of subsamples taken, where the latter scales linearly.

The rest of the paper is organized as the following. In Materials and Methods, we explain the proposed bootstrap subsampling procedure, highlight important considerations for some of the steps, and elaborate on potential use cases. In Results, some of the proposed use cases (model selection and goodness of fit) are demonstrated with simulated and empirical data. Lastly, we conclude with Discussion.

## Materials and Methods

### Subsampling scheme and resampling distributions

Each subsample of the bootstrap subsampling scheme of Bhattacharyya *et al*.^28^ consists of a uniform node-wise subsample of all the nodes in the observed network *G*_*o*_ (with node set *V*_*o*_ and edge set *E*_*o*_) and their induced subgraph, i.e., the nodes in the subsample and all edges between these nodes. For each subsample, one may compute any set of statistics to form a resampling distribution of these statistics. Although the subsamples will not have the same properties as the full network or a network of the same size as the subsample drawn from the true data generating mechanism^32^, they will still retain features of the true data generating mechanism since the subsampling does not change any between-edge or between-node dependence that influenced the formation of the network, despite adding a degree of “missingness” by removing elements correlated with those in the subsample. In comparison, should one generate draws from a particular fitted model to form a resampling distribution, the between-edge and between-node dependencies will be those specified by the fitted model; in this case, the generated networks will only be representative of the true data generating mechanism if the fitted model is the true model, which is a strong assumption and usually not verifiable in practice.

**Figure 1:**
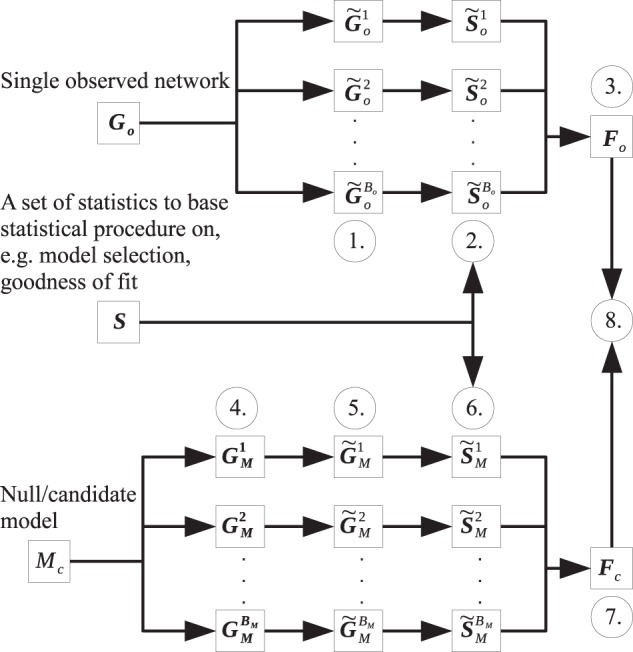
Schematic of the steps of the proposed bootstrap subsampling procedure for a single observed network *G*_*o*_: 1. Obtain subgraphs induced by *B*_*o*_ subsamples of the nodes of **G**_*o*_. 2. Compute the chosen network statistics **S** for each induced subgraph. 3. Form resampling distribution *F*_*o*_ of **S** from ${\tilde{{\bf{S}}}}_{o}^{1}\ldots {\tilde{{\bf{S}}}}_{o}^{{B}_{o}}$. 4. Draw networks of same size as **G**_*o*_ from network model *M*_*c*_. 5. For each generated network **G**_*M*_^1^ … **G**_*M*_^*B*^*M*, obtain one subgraph induced by one subsample of the nodes of each network. 6. Compute **S** for each induced subgraph (${\tilde{{\bf{S}}}}_{M}^{1}\ldots {\tilde{{\bf{S}}}}_{M}^{{B}_{M}}$). 7. Form resampling distribution *F*_*c*_ of **S** from ${\tilde{{\bf{S}}}}_{M}^{1}\ldots {\tilde{{\bf{S}}}}_{M}^{{B}_{M}}$. 8. Perform statistical procedure by comparing *F*_*o*_ and *F*_*c*_.

Because each subsample only consists of a subset of *V*_*o*_ and *E*_*o*_, each subsample will be missing elements that are correlated with those that are included in the subsample. As a result, this must be taken into account when any comparisons are made with a null/candidate model *M*_*c*_. One may be tempted to compare subsamples of *G*_*o*_ with draws from *M*_*c*_ of the same size as the subsamples. This should however be avoided since there is a degree of “missingness” in the subsamples of *G*_*o*_ that are not present in such draws from *M*_*c*_. Even if *M*_*c*_ was the true model, this disparity could make the two behave differently. Instead, one should generate draws from *M*_*c*_ of the same size as *G*_*o*_ and then apply the same subsampling scheme to these draws. This ensures that both the subsamples of *G*_*o*_ and those of *M*_*c*_ have the same amount of “missingness” and are comparable. Should *M*_*c*_ be representative of the true data generating mechanism, then the behavior of the two subsamples their corresponding resampling distributions of computed statistics should be similar. The representativeness of the subsamples from *G*_*o*_, as well as their comparability with the subsamples from *M*_*c*_, form the basis of our procedure. Even though we only consider uniform subsampling, the subsampling method is flexible and can be chosen to be representative of sampling in practice or for statistical and computational ease. The proposed bootstrap subsampling procedure is summarized in Fig. 1.

In contrast to existing methods that also use draws from the fitted model to assess goodness of fit, this approach can lead to a richer comparison. For existing methods, after choosing the statistics for assessing goodness of fit, the given statistics are computed for *G*_*o*_ and for a large number of draws from *M*_*c*_. The point estimate of these statistics for *G*_*o*_ are then placed within the distribution of said statistics of the draws from *M*_*c*_. Goodness of fit is assessed by the location of the point estimate from *G*_*o*_ within the draws from *M*_*c*_. This can be done visually or by quantifying the proportion of the draws with values of the statistics deemed more extreme. With our approach, the two resampling distributions can be compared in many ways, such as their location, spread, and shape. In addition, one can quantify the distance between the two distributions using, for example, the Kolmogorov-Smirnov (KS) statistic (defined for discrete distributions also^33^) or the Kullback-Leibler divergence to order the fit of different candidate models.

One point of interest and emphasis is that the subsamples from *G*_*o*_ are all from a single network, while the subsamples from *M*_*c*_ are subsamples of independent network realizations drawn from *M*_*c*_ instead of subsamples from a single network drawn from *M*_*c*_. This scheme is proposed due to potential instability of single generated networks and the corresponding subsamples, since there can be a great deal of instability in the generated networks depending on the model, including the seed network used to grow networks specified by mechanistic models. In addition, the disparity between the two types of subsamples may depend on the proportion of the nodes in each subsample. Both of these points are important to the performance of the procedure and are further examined in the next two sections.

### Stability under sampling

When sampling from the candidate model, one needs to take care to ensure that the draws from *M*_*c*_ behave like the observed network even if the candidate model is the true model or an accurate model, and in turn, the subsamples of these draws behave like the subsamples of the observed network. In the worst case, such draws can look nothing like the observed network despite using a good candidate model, e.g., the draws could have highly varying degree distributions that look nothing like that of the observed network. This issue can be more prominently demonstrated in the context of some mechanistic network models.

Networks generated from mechanistic models are often grown from a small (relative to the final size of the network) seed network according to the model’s generative mechanism until some stopping condition is reached, e.g., attaining a requisite number of nodes. There is research showing that the original seed network has no influence on the degree distribution in the limit, i.e., for a large number of nodes, for certain types of mechanistic network models^34,35^. While some data sets, such as online social networks, may be sufficiently large to reach this asymptotic regime, others, such as protein-protein interaction networks, may not be. Thus, when generating draws from candidate models for analysis of smaller networks, the original seed network can potentially have a great deal of influence. The seed network maybe as simple as a single node, or a complete graph of only three nodes, up to bigger complete graphs, or something more elaborate with more than one component. We briefly examine the effect of the seed network on the stability of the degree distribution of networks generated from the Erdös-Rènyi and duplication-divergence models, which are frequently used to model protein-protein interaction networks.

#### Erdös-rènyi model

The Erdös-Rènyi (ER) model^36^ is a simple but rather unique model in that it can be framed as both a mechanistic and a statistical model. In the ER model, the number of nodes *n* is fixed, and there are two variants of the model that determine how the edges are placed. In the first variant, the *G*(*n*,*p*) model, each of the *C*(*n*, 2), *n* choose 2, possible edges are independent and are included in the graph with probability *p*, so the number of edges in the graph is binomial. In the other variant, the *G*(*n*,*m*) model, the number of edges in the graph *m* is also fixed. In this case, the random graph has a uniform distribution over all *C*(*C*(*n*, 2),*m*) possible graphs with *n* nodes and *m* edges.

The first variant can be easily framed as a mechanistic model. The network generation starts with a seed network of a single node. Then at each stage, a new node is added, and an edge between the new node and each existing node is added with probability *p*. This is done until there are *n* nodes in the network. Rather than starting with a seed network of a single node, networks can be generated according to the generative mechanism of the *G*(*n*,*p*) model initialized with a different seed network. Here, we generated *G*(*n* = 1000, *p* = 0.1) networks according to these rules, with complete graphs of 5, 8, 10, 20, 50, 100 nodes as the seed networks. We generated 50 networks of each size of the seed to evaluate the influence of the seed network on the stability of the degree distribution of the fully grown network.

**Figure 2:**
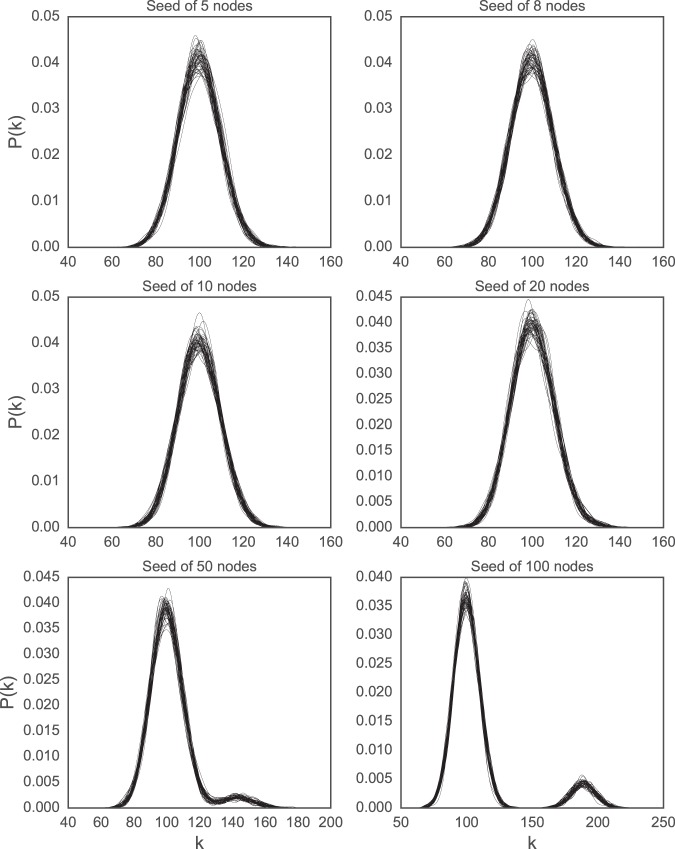
The degree distribution of 50 generated graphs from the *G*(*n* = 1000, *p* = 0.1) model with seeds of 5, 8, 10, 20, 50, 100 nodes, from left to right, then top to bottom, as described in text. This and the next figure show the differing influence the seed can have on variability.

The degree distribution of the 50 generated graphs at each size of the seed network are plotted in Fig. 2. While the shape of the degree distribution understandably changes as the complete graph used as the seed network gets bigger, the size of the seed network seems to have little influence on the stability of the degree distribution. All 50 networks, for each size of the seed network, have very similar degree distributions. The width of the “band” of the 50 distributions stacked on top of one another also looks to be mostly unchanging. This seems to indicate that the variability in the degree distribution is largely unaffected by the size of the seed network.

#### Duplication-divergence models

Duplication-divergence models are a popular class of models used for protein-protein interaction networks. Some examples include the duplication-mutation-complementation (DMC)^18^ and duplication-mutation-random mutation models (DMR)^17,37^. Given a seed network, both DMC and DMR models grow the network according to their respective generative mechanisms until the requisite number of nodes, *n*, is reached. In both the DMC and DMR models, a new node is first added at the beginning of each step in network generation. An existing node is chosen uniformly at random for duplication, and an edge is then added between the new node and each neighbor of the chosen node. After this, the two models diverge. For DMC, for each neighbor of the chosen node, one of the edge between the chosen node and the neighbor or the edge between the new node and the neighbor is randomly chosen and then removed with probability *q*_*mod*_. The step is concluded by adding an edge between the chosen node and the new node with probability *q*_*con*_. For DMR, each edge connected to the new node is removed independently with probability *q*_*del*_. The step concludes by adding an edge between the new node and any existing node at the start of step *t* with probability *q*_*new*_/*n*(*t*), where *n*(*t*) is the number of nodes in the network at the start of step *t*.

**Figure 3:**
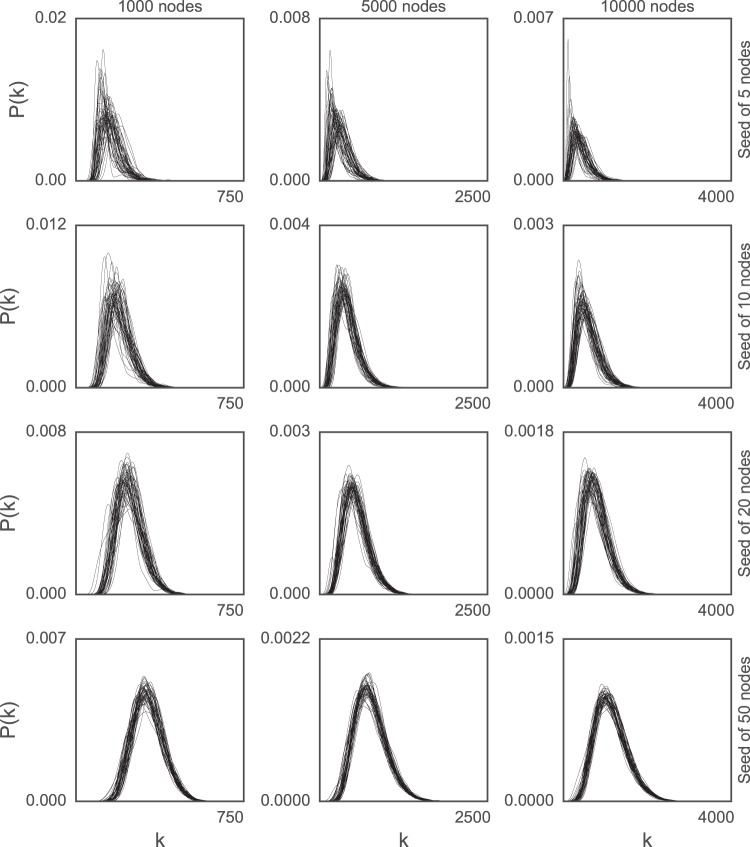
Degree distribution of 50 generated graphs of 1000, 5000, 10000 nodes from the DMC model, left to right, with seeds of 5, 10, 20, 50 nodes, top to bottom. This and the previous figure show the differing influence the seed can have on variability.

To assess the stability of the degree distribution, we generated 50 network realizations of 1000, 3000, 5000, 7000, 10000 nodes from both models with the seed network set as a complete graph with 5, 8, 10, 20, 50, 100 nodes. The parameters of the DMC model were set as *q*_*mod*_ = 0.2 and *q*_*con*_ = 0.1, while those of the DMR model were *q*_*del*_ = 0.2 and *q*_*new*_ = 0.1. The degree distribution for the 50 generated DMC networks for a subset of all combinations of the size of the seed network and the total number of nodes are plotted in Fig. 3; those for all combinations for both DMC and DMR models can be found in the Supplementary Information (Figs S1 and S2). A general trend in the plots is that the total number of nodes in the network has little to no influence on the stability of the degree distribution, while the size of the seed network has a great deal of influence, with stability increasing sharply with the size of the seed network, up to 50. For smaller seed networks (5 nodes), the shape and spread of the degree distributions vary wildly even for larger networks. For a modest increase in the size of the seed network (10 nodes), the shape and the spread of the degree distributions become more similar. Finally, for larger seed networks (20 or 50), the shape and spread of the degree distributions are quite uniform, and the width of the “band” of the 50 degree distributions stacked on top of one another also decreases. Clearly, the variability of the degree distribution depends greatly on the size of the seed network.

One important difference between the ER and DMC/DMR models is the dependence on exisiting edges on the formation of new ones. The instability in the degree distribution of networks generated from DMC/DMR models with small seed networks can be attributed to this dependence. While these two examples show the influence the seed network can potentially have in generating networks of modest size with mechanistic models, it does beg the question of how one selects a meaningful seed that leads to stable sampling while mimicking the behavior of the observed network in a principled way. Hypothetically, if the observed network is indeed generated from an ER model and assuming the seed network and the parameter values are well chosen, then the generated networks should mostly appear similar to the observed network due to the low variability regardless of the size of the seed. On the other hand, should the observed network come from a DMC/DMR model and assuming well chosen parameter values, as well as an appropriate but small seed network, then the generated networks are unlikely to appear similar to the observed network due to the high variability with small seeds as demonstrated.

### Portion of nodes to include in subsamples

The portion of nodes included in each subsample should not be so small such that no characteristics of the observed network or candidate models are retained, but also not so big such that the subsamples contain little variability. In one extreme, each subsample consists of just one node so that there is no structure within the induced subgraph, and in the other extreme, each subsample is simply the entire network. While the latter is of little concern when taking subsamples from independent draws from candidate models, it leaves no variability in the subsamples from a single observed network such that any resulting resampling distribution would simply be a point mass. We investigate what is an appropriate portion of nodes to include in each subsample through a detailed example with one particular model. The details can be found in the Supplementary Information.

In our example, we define the criterion for performance in terms of the expectation of the KS statistic (smaller values are better) between *F*_1_, the resampling distribution from the subsamples of a single network drawn from candidate model *M*_*c*_, and *F*_*c*_, the resampling distribution from subsamples of several independent networks drawn from *M*_*c*_, where each subsample comes from a different independent draw. This quantity is a measure of how closely *F*_*o*_, the resampling distribution from the subsamples of the observed network, matches *F*_*c*_ when the observed network is truly generated by *M*_*c*_. If the KS statistic is small, discrepency between *F*_*o*_ and *F*_*c*_ will be small if the model is correct. Additionally, this quantity being small implies that there is not much difference between using *F*_1_ or *F*_*c*_ for comparison with *F*_*o*_, thus we would be better off in electing for the stability of *F*_*c*_. Note that the computation time required for *F*_*c*_ is greater than that for *F*_1_. Although not completely generalizable, our example suggests to keep the portion of nodes in the subsample low (<30% in this example) as long as enough features of the models can be retained.

### Proposed use cases

There are a variety of statistical procedures that can take advantage of this sampling scheme, with a few of them detailed below. Before proposing the general framework for a few typical statistical procedures via the bootstrap subsampling procedure, we define the following notation for the rest of the section. The observed network will be referred to as *G*_*o*_ with *B*_*o*_ subsamples and corresponding induced subgraphs ${\tilde{G}}_{o}^{(1)}\ldots {\tilde{G}}_{o}^{({B}_{o})}$. The draws from candidate model *M*_*c*_ will be referred to as *G*_*M*_^1^ … *G*_*M*_^*B*^*M* with corresponding subsample induced subgraphs ${\tilde{G}}_{M}^{(1)}\ldots {\tilde{G}}_{M}^{({B}_{M})}$. Given a set of network statistics *S* chosen for model selection or assessing goodness of fit, the set computed from ${\tilde{G}}_{o}^{(1)}\ldots {\tilde{G}}_{o}^{({B}_{o})}$ will be referred to as ${\tilde{S}}_{o}^{(1)}\ldots {\tilde{S}}_{o}^{({B}_{o})}$, while those computed from ${\tilde{G}}_{M}^{(1)}\ldots {\tilde{G}}_{M}^{({B}_{M})}$ will be referred to as ${\tilde{S}}_{M}^{(1)}\ldots {\tilde{S}}_{M}^{({B}_{M})}$. Note that *B*_*o*_ and *B*_*M*_ need not be equal.

#### Model selection

Suppose the goal is to select between candidate models *M*_1_ … *M*_*c*_ for *G*_*o*_. Given a set of statistics *S* to base the model selection on, one needs to compute ${\tilde{S}}_{{M}_{i}}^{(1)}\ldots {\tilde{S}}_{{M}_{i}}^{({B}_{M})}$ from ${\tilde{G}}_{{M}_{i}}^{(1)}\ldots {\tilde{G}}_{{M}_{i}}^{({B}_{M})}$ for *i* = 1 … *c*. These collections of statistics along with the model indices of each draw form the training data and are the basis for the model selection procedure. The selection of *S* is flexible and should be chosen to prioritize the aspects of the network where similarity to the observed network is most paramount. The training data can be used to train any learning algorithm for prediction of the model index. Examples include random forest, support vector machine, and ensemble learning algorithms like the Super Learner^38–40^. The trained algorithm is evaluated at each of ${\tilde{S}}_{o}^{(1)}\ldots {\tilde{S}}_{o}^{({B}_{o})}$ to give selected model ${\hat{M}}_{1}$ … ${\hat{M}}_{{B}_{o}}$, with majority rule deciding the final selected model.

**Algorithm 1:**
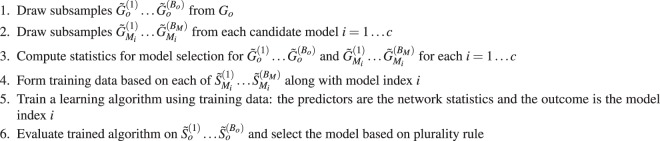
Steps for the model selection with the bootstrap subsampling procedure.

One distinct advantage of the model selection through this bootstrap subsampling procedure is that it gives inherent evidence about uncertainty or confidence in the selected model as well as other candidate models. The proportion of ${\tilde{G}}_{o}^{(1)}\ldots {\tilde{G}}_{o}^{({B}_{o})}$ that are assigned to each model can be seen as evidence in favor of each candidate model, while the proportion of subsamples assigned the model that forms the majority can be seen as confidence in the selected model. With algorithms like random forest, where the decision is based on plurality rule, this aspect of our approach does not add anything new. But with others, such as support vector machine or the Super Learner that are not based on plurality rule, this approach offers a way to quantify uncertainty without the need to alter the learning algorithm itself.

#### Goodness of fit

To assess the goodness of fit for candidate models *M*_1_ … *M*_*c*_, the procedure is similar to that of model selection. For a set of statistics *S* for assessing goodness of fit, one computes ${\tilde{S}}_{o}^{(1)}\ldots {\tilde{S}}_{o}^{({B}_{o})}$ from ${\tilde{G}}_{o}^{(1)}\ldots {\tilde{G}}_{o}^{({B}_{o})}$ and ${\tilde{S}}_{{M}_{i}}^{(1)}\ldots {\tilde{S}}_{{M}_{i}}^{({B}_{M})}$ from ${\tilde{G}}_{{M}_{i}}^{(1)}\ldots {\tilde{G}}_{{M}_{i}}^{({B}_{M})}$ for *i* = 1 … *c*. Rather than training a learning algorithm based on ${\tilde{S}}_{{M}_{i}}^{(1)}\ldots {\tilde{S}}_{{M}_{i}}^{({B}_{M})}$ as in model selection, ${\tilde{S}}_{o}^{(1)}\ldots {\tilde{S}}_{o}^{({B}_{o})}$ are directly compared against ${\tilde{S}}_{{M}_{i}}^{(1)}\ldots {\tilde{S}}_{{M}_{i}}^{({B}_{M})}$ for each *i* to assess fit. As mentioned above, this comparison between the distribution of ${\tilde{S}}_{o}^{(1)}\ldots {\tilde{S}}_{o}^{({B}_{o})}$ and any set of ${\tilde{S}}_{{M}_{i}}^{(1)}\ldots {\tilde{S}}_{{M}_{i}}^{({B}_{M})}$ can be done in terms of location, spread, shape, or other aspects of the distribution.

**Algorithm 2:**
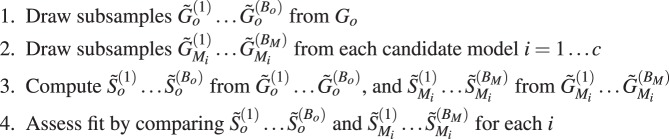
Steps for assessing goodness of fit with the bootstrap subsampling procedure.

Assessment based on any one of these aspects may however lead to conflicting results, i.e., different models having the best fit depending on which aspect the comparison is based on, and it might be desirable to make comparisons through a more holistic measure. One solution to this is to compute a distance measure, such as the KS statistic or the Kullback-Leibler divergence, between ${\tilde{S}}_{o}^{(1)}\ldots {\tilde{S}}_{o}^{({B}_{o})}$ and ${\tilde{S}}_{{M}_{i}}^{(1)}\ldots {\tilde{S}}_{{M}_{i}}^{({B}_{M})}$ to quantify the fit of model *i*. This gives a single statistic that takes the entire distribution into account to quantify and to categorically order the fit of each candidate model. The KS test statistic and Kullback-Leibler divergence are typically computed in one dimension and can be used to compare the fit for each statistic individually as is. Instead, should one wish to make a comparison based on all statistics *S* at the same time, one can look to use generalizations of these statistics^41–43^.

#### Comparison of multiple networks

If multiple networks are observed instead of a single network, and the goal is to assess how similar they are, then one can do so by building a resampling distribution from multiple networks. For the case of two observed networks with a set of statistics *S* for comparison and observed networks *G*_*o*1_ and *G*_*o*2_, one can compute ${\tilde{S}}_{o1}^{(1)}\ldots {\tilde{S}}_{o1}^{({B}_{o1})}$ and ${\tilde{S}}_{o2}^{(1)}\ldots {\tilde{S}}_{o2}^{({B}_{o2})}$ from subsamples ${\tilde{G}}_{o1}^{(1)}\ldots {\tilde{G}}_{o1}^{({B}_{o1})}$ and ${\tilde{G}}_{o2}^{(1)}\ldots {\tilde{G}}_{o2}^{({B}_{o2})}$, respectively. The comparison of the two is based on ${\tilde{S}}_{o1}^{(1)}\ldots {\tilde{S}}_{o1}^{({B}_{o1})}$ and ${\tilde{S}}_{o2}^{(1)}\ldots {\tilde{S}}_{o2}^{({B}_{o2})}$, and one can proceed essentially the same way as with goodness of fit by comparing different aspects of the two distributions, but with ${\tilde{S}}_{o1}^{(1)}\ldots {\tilde{S}}_{o1}^{({B}_{o1})}$ and ${\tilde{S}}_{o2}^{(1)}\ldots {\tilde{S}}_{o2}^{({B}_{o2})}$ in place of ${\tilde{S}}_{o}^{(1)}\ldots {\tilde{S}}_{o}^{({B}_{o})}$ and ${\tilde{S}}_{{M}_{i}}^{(1)}\ldots {\tilde{S}}_{{M}_{i}}^{({B}_{M})}$. Should there be more than two observed networks for comparison, then the distance measure statistics can once again be used to quantify all pairwise relative similarities between the observed networks.

## Results

### Simulation and empirical data

We use simulation studies as well as data from an empirical network to illustrate the use of the bootstrap subsampling procedure in some of the scenarios described in the previous section. The simulated data and all code can be found under the Supplementary Information, while the protein-protein interaction data can be downloaded from the database of interacting proteins (DIP)^44^ website directly.

#### Model selection

The simulation studies conducted for model selection consider instances of a variation on the afformentioned *G*(*n*,*m*) model we introduced^40^. This variation generates random graphs with *n* nodes and *m* edges just as the *G*(*n*,*m*) model with each edge being added one at a time. At each step in network generation, a pair of unconnected nodes are selected at random, and the probability for adding an edge between the two is determined based on the number of triangles it would close; the edge is then added with the given probability. This is repeated until there are *m* edges in the network. If the probability for adding an edge is fixed, then this is the *G*(*n*,*m*) model. Instead, we start with a base probability *p*_0_ to add the edge. Should the edge close at least one triangle, the probability increases by *p*_1_. Should multiple triangles be closed by the edge, then the probability further increases by *p*_Δ_ for each additional triangle closed.

In the simulation, we select between two instances of this model, both having *p*_0_ = 0.3 and *p*_1_ = 0.1. The difference comes in *p*_Δ_, with *p*_Δ_ = 0 for model 1, while *p*_Δ_ varies over 0.05, 0.03, 0.01, 0.005 for model 2. For a given choice of *n* and *m*, as *p*_Δ_ decreases and gets closer to 0, the difference between the two models becomes more difficult to detect. The generated networks from both models consist of 100 nodes with edge count varying over 100, 500, 1000, 2000. This gives a total of 20 comparisons between the models, one for each combination of values of *p*_Δ_ and *m*. For a given set of parameter values, the difference between the two models should be easier to detect as edge count increases, since the difference due to *p*_Δ_ has more opportunities to manifest itself. The training data consists of a single subsample of 80 nodes for each of 10000 draws from each model (${\tilde{G}}_{{M}_{i}}^{(1)}\ldots {\tilde{G}}_{{M}_{i}}^{(10000)}$), where *i* = 1, 2. The test data consists of 1000 draws from each model (*G*_*o*_), while the model selection is based on 100 subsamples of 80 nodes from each draw (${\tilde{G}}_{o}^{(1)}\ldots {\tilde{G}}_{o}^{(100)}$). Although 100 nodes seems few, it is already large enough for a network to give rise to a very large resampling distribution. Additionally, despite the simplicity of the model we are using, 100 nodes is large enough for the likelihood function to be intractable.

The model selection is through the Super Learner^38–40^, with support vector machine (*ν*-classification with *ν* = 0.5, radial kernel), random forest (*N*_*tree*_ = 1000, min terminal node size = 1), and *k*-nearest neighbors (*k* = 10) as candidate algorithms, and average clustering coefficient, triangle count, and the three quartiles of the degree distribution as predictors. Note the parameters for the candidate algorithms are in parentheses. These statistics were chosen as predictors since the difference in *p*_Δ_ directly affects formation of triangles, while the other statistics are influenced strongly by triangles. For each of the 100 ${\tilde{G}}_{o}^{({b}_{o})}$ for a particular testing network *G*_*o*_, the Super Learner will give a score between 0 and 1 for predicting the model class of ${\tilde{G}}_{o}^{({b}_{o})}$, with score < 0.5 assigned model 1 and score >0.5 assigned model 2. The selected model is the model assigned to ${\tilde{G}}_{o}^{({b}_{o})}\,$s more frequently.

**Figure 4:**
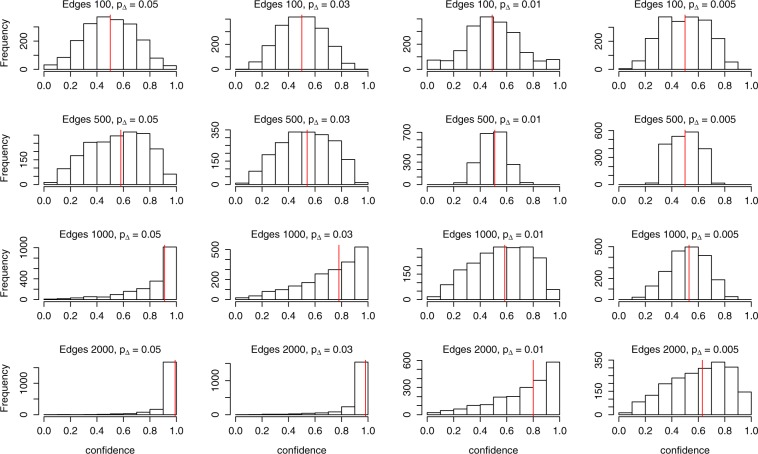
Histograms of the confidence score (proportion of subsamples assigned the correct model here rather than the majority) for *p*_Δ_ from 0.05, 0.03, 0.01, 0.005, from left to right, and edge count from 100, 500, 1000, 2000, from top to bottom, with the red vertical lines representing the median. This shows that our proposed approach for model selection behaves as one would intuitively expect, i.e., greater differences between the models are more frequently classified correctly than smaller differences.

The results of the simulation are summarized in Fig. 4 and Table 1. Table 1 contains the proportion of test networks whose model was correctly classified by the Super Learner at each combination of *p*_Δ_ and edge count. Unsurprisingly, the proportion decreases as *p*_Δ_ decreases for a fixed edge count, and increases as edge count increases for a fixed *p*_Δ_. Figure 4 shows the histogram of the confidence for the correct model. When model 1 is the true model of the test network, this is the proportion of the 100 subsamples that were assigned model 1, and vice versa. When the proportion of correctly classified models is around 0.5, i.e., as good as a random guess, the confidence is symmetric and centered close to 0.5. When the proportion is higher than 0.5, the distribution of the confidence is shifted to the right, meaning that the two models are easier to tell apart. In addition, the more right skewed the histograms, the more confidence in the correct model. The red vertical line indicates the median, which also moves to the right as the proportion increases and as the confidence becomes more right skewed. This behavior indicates that the confidence for the selected model from the bootstrap subsampling procedure quantifies well the degree of uncertainty in the selected model. Random forest feature importance of all five predictors can be found in the Supplementary Information (Fig. S3) to see the shifting role of the predictors in the different scenarios.

**Table 1:** Proportion of the test networks correctly classified at each combination of *p*_Δ_ and edge count.

	*p*_Δ_ = 0.05	0.03	0.01	0.005
Edge count = 100	0.5015	0.5005	0.4834	0.5100
500	0.6092	0.5670	0.5178	0.5076
1000	0.9203	0.8202	0.6249	0.5786
2000	0.9890	0.9740	0.8343	0.6810

#### Goodness of fit

To display our method for assessment of goodness of fit, we examine the yeast (*S.cerevisiae*) protein-protein interaction network data from DIP^44^. This data set has been much examined in the literature, including using network models. There are two particular publications^45,46^ that fit different duplication divergence models to two different previous versions of the yeast data set, with differing seed networks. Here we apply our method to compare the fit of the two different models on the most recent version of the data.

Both papers use the same duplication divergence model^17,37^, which we described as DMR earlier. However, the papers used different parameter values and different seed networks. The fit from Hormozdiari *et al*.^45^ has parameter values *p* = 0.365 and *r* = 0.12, and the seed network contains 50 nodes. The seed network was constructed by highly connecting cliques, complete graphs where an edge exists between every pair of nodes, of 7 nodes and 10 nodes, then connecting additional nodes to the cliques. To highly connect the cliques, each possible edge between nodes in different cliques (70 such edges) was added with probability 0.67. Then, another 33 nodes were attached to randomly chosen nodes from the two cliques. At each step of the network generation, if a singleton (a node not connected to any other node) was generated, it was immediately removed in their model. Note that the details for obtaining the seed network from Hormozdiari *et al*.^45^ were somewhat incomplete, so this is our interpretation of the description of their seed network.

On the other hand, the fit from Schweiger *et al*.^46^ has parameter values *p* = 0.3 and *r* = 1.05. The authors use a smaller seed network of 40 nodes, generated with an inverse geometric model. To generate this seed network, a set of coordinates {*x*_1_ … *x*_40_} in ℝ^*d*^ is generated for each node. Then, each pair of nodes with distance *x*_*i*_ − *x*_*j*_ greater than some threshold *R* is connected with an edge. Each dimension of the coordinates is independently generated from the standard normal distribution *N*(0, 1). In their fit, the seed network uses *d* = 2 and *R* = 1.5. Unlike Hormozdiari *et al*.^45^, Schweiger *et al*.^46^ does not remove singletons as they are generated.

Both papers assessed the fit of their model by comparing certain aspects of the generated network to those of the yeast PPI network. In Hormozdiari *et al*.^45^, model fit was assess via *k*-hop reachability, the number of distinct nodes reachable in ≤*k* edges, the distribution of particular subraphs, such as triangles and stars, as well as some measures of centrality. Schweiger *et al*.^46^ assess fit with the distribution of bicliques, i.e., subgraphs of two disjoint sets of nodes where every possible edge between the two sets exists. Here, we assess the fit of both models via our method with the average local clustering coefficient^16^, triangle count, and the degree assortativity^47^. The local clustering coefficient of a particular node is a measure of to what extent its neighbors resemble a clique. Mathematically, this is computed as the number of edges between a node’s neighbors divided by the maximum possible number of such edges. We use the average of the local clustering coefficient over all nodes in the network as a meassure of local clustering that is also attributable to the network as a whole. We also consider the number of triangle subgraphs that appear in the network. Unlike Hormozdiari *et al*.^45^, which counts the total number of various subgraphs together, the count of triangles alone is a strictly global measure of clustering. Lastly, the degree assortativity of a network is a measure of how similar are the degrees of nodes connected by an edge. It is defined as the Pearson correlation of the degrees of nodes connected by an edge, so positively assorted networks have more edges between nodes of similar degrees, while negatively assorted networks have more edges between nodes of dissimilar degrees.

For the analysis, we consider the largest connected component (LCC) of the PPI network just as in Hormozdiari *et al*.^45^. The full network from the current version of the data contains 5176 nodes and 22977 edges, while the LCC contains 5106 (98.6%) nodes and 22935 (99.8%) edges. Networks drawn from each model contain the same number of nodes as the LCC, starting from their respective seed networks described above. Subsamples from the PPI network as well as networks drawn from each model contain 1550 nodes, roughly corresponding to 30%. This was the largest portion considered in our study of portion of nodes subsampled above.

**Figure 5:**
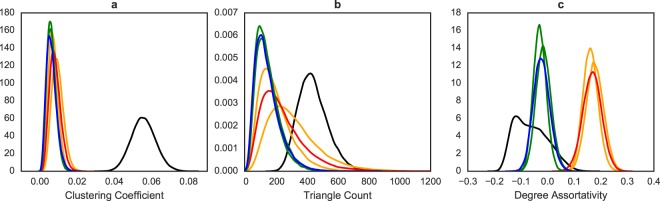
The *resampling* distribution of clustering coefficient (panel a), triangle count (panel b), and degree assortativity (panel c) from independent draws from the two model fits (blue for Hormozdiari *et al*.^45^ and red is for Schweiger *et al*.^46^) as well as the PPI network (black). In addition, there are two resampling distributions from a single draw from each of the two model fits (green for Hormozdiari *et al*.^45^ and orange is for Schweiger *et al*.^46^). This figure gives a visual representation of the additional information provided by the goodness of fit approach as well as difference from comparing point estimates with distribution of the statistics from full networks as seen in Fig. 6.

The results of the data analysis are summarized in Fig. 5, where it is clear that the ordering of the fit of both models differs based on the network statistic of comparison. In accordance with earlier notation, for each statistic, we refer to the resampling distribution of the model of Hormozdiari *et al*.^45^ as *F*_*c*_^*h*^ and that of Schweiger *et al*.^46^ as *F*_*c*_^*s*^, while that of the PPI network is referred to as *F*_*o*_.

For clustering coefficient (Fig. 5a), both models fit equally poorly, as neither *F*_*c*_^*h*^ nor *F*_*c*_^*s*^ have any overlap with *F*_*o*_. The KS statistic between *F*_*o*_ and each of *F*_*c*_^*h*^ and *F*_*c*_^*s*^ are both 1, indicating very poor fit. For triangle count (Fig. 5b), the model of Schweiger *et al*.^46^ seems to fit better as *F*_*c*_^*s*^’s spread has a much bigger overlap with *F*_*o*_. The KS statistic between *F*_*o*_ and *F*_*c*_^*s*^ (0.6778) is also much smaller than that between *F*_*o*_ and *F*_*c*_^*h*^ (0.9018). Lastly, for degree assortativity (Fig. 5c), the model of Hormozdiari *et al*.^45^ fits much better as the spread of *F*_*c*_^*h*^ overlaps with that of *F*_*o*_, and most of *F*_*c*_^*h*^’s spread is negative just as *F*_*o*_. On the other hand, *F*_*c*_^*s*^ is entirely positive and has little overlap with *F*_*o*_. The KS statistic tells the same story, with 0.4373 for Hormozdiari *et al*.^45^ and 0.9782 for Schweiger *et al*.^46^.

**Figure 6:**
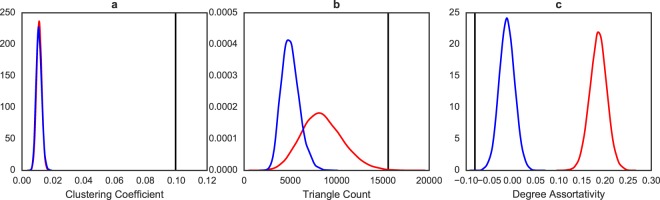
The distribution of clustering coefficient (panel a), triangle count (panel b), and degree assortativity (panel c) from independent *full network* draws from the two model fits (blue for Hormozdiari *et al*.^45^ and red is for Schweiger *et al*.^46^). The corresponding point estimates from the *full* PPI network are the vertical black lines.

In, Fig. 6, we plot the distribution of the same statistics from full network realizations drawn from the two models, as well as the point estimate from the full PPI network. We use *L*_*c*_^*h*^ and *L*_*c*_^*s*^ as the full network analogs to *F*_*c*_^*h*^ and *F*_*c*_^*s*^, respectively, and *S*_*o*_ to denote the point estimate for the full PPI network. For clustering coefficient, *L*_*c*_^*h*^ and *L*_*c*_^*s*^ look very similar, so this comparison would not lead to a different conclusion. For triangle count, *L*_*c*_^*s*^ visually appears somewhat closer to *S*_*o*_ than *L*_*c*_^*h*^. The spread of *L*_*c*_^*s*^ also contains *S*_*o*_, albeit barely. However, *L*_*c*_^*s*^ is also much more variable than *L*_*c*_^*h*^. In fact, *L*_*c*_^*s*^’s spread reaches farther than that of *L*_*c*_^*h*^ on both ends. Based on *L*_*c*_^*h*^, *L*_*c*_^*s*^ and *S*_*o*_, it is not obvious which model fits better, whereas our method gives a clear numerical ordering between the two models. For degree assortativity, the entirety of *L*_*c*_^*h*^ is closer to *S*_*o*_ than *L*_*c*_^*s*^, so this comparison would not lead to a different conclusion just as clustering coefficient. Finally, since our method provides a joint distribution of the three statistics from each model as well as the PPI network, we are able to quantify overall fit that takes all three statistics into account jointly via a distance between the joint distributions (such as the multidimensional KS statistic as discussed earlier). This example demonstrates that considering the full resampling distributions, rather than point estimates as existing methods do, results in a more nuanced comparison of network models with empirical data.

Additionally, in Fig. 5, we plot the subsamples from two individual networks drawn from each model against the subsamples from independent networks drawn from each model. For each statistic, the spread and location of the two types of subsamples are similar, although triangle count shows a little more deviation than the other two since it is a sum rather than a mean. This is likely due to the rather large seeds (50 and 40 nodes) both models use as well as the rather small portion of nodes in each subsample (~30%), reflecting our observations in earlier sections.

## Discussion

Network models are able to model increasingly complex dependencies that arise in network data. Yet this very dependency poses a statistical challenge, especially in the case of a single observed network. We propose a bootstrap subsampling procedure as a basis for statistical procedures in this setting that is based on a flexible resampling distribution built from the single observed network and demonstrate the procedure in both simulation and empirical test settings.

Given any network statistic of interest, its resampling distribution from the observed network can be compared against its analog from a null/candidate model based on any attribute of the distributions, such as location, spread, shape, measures of mean, and through pairwise distances. In comparison, existing methods in this setting typically rely on the point estimate from the observed network, which leads to a more limited comparison. As seen in our empirical example, this additional layer of information can sometimes lead to a different conclusion than existing methods. In addition, the distance between the resampling distributions serves as an overall measure for comparison and provides an ordering of different network models.

The flexibility of our approach is not limited to what one can do with the resampling distributions, but also extends to the type of subsampling used to generate them. Although here we used simple random samples of the nodes of the network, other schemes are possible. In fact, any method of subsampling is valid as long as it is applied to both the observed and model generated data. Thus, it can be tailored to the needs of the investigator, including statistical or computational considerations. The method of subsampling can be also used as a sensitivity analysis to see whether the results of the analysis remain unchanged under different methods of subsampling. This consideration for different methods of subsampling motivates the most immediate step for future work as it begs the question whether they can lead to performance gains. Perhaps certain types of subsampling schemes can outperform others given the method of sampling used to obtain the observed data.

## Supplementary information

Supplementary information

Code and data
