# Supplementary material for: A Bootstrap Method for Goodness of Fit and Model Selection with a Single Observed Network
Source: Sci Rep. 2019 Nov 13;9:16674. doi: 10.1038/s41598-019-53166-6 (PMC6854093; doi:10.1038/s41598-019-53166-6)
Supplement: Supplementary file 1 — Supplementary information [file 41598_2019_53166_MOESM1_ESM.pdf]

A Bootstrap Method for Goodness of Fit and Model Selection with a Single Observed Network: Supplementary Information

Sixing Chen[1] and Jukka-Pekka Onnela[1]

[a]Department of Biostatistics, Harvard T.H. Chan School of Public Health

655 Huntington Ave, Boston, MA 02115

sixingchen@hsph.harvard.edu, onnela@hsph.harvard.edu

Additional Figures

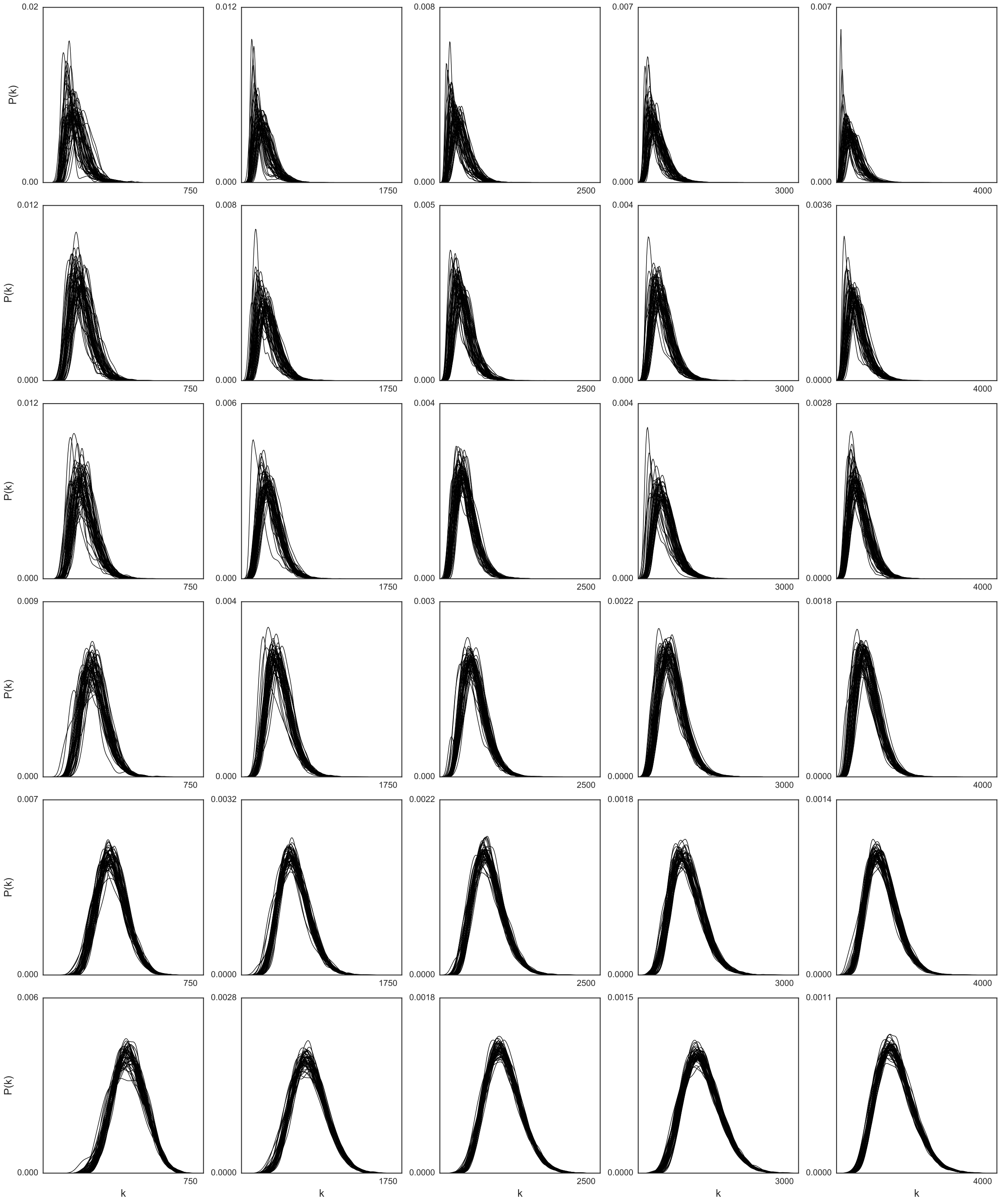

Figure S1: The degree distribution of 50 generated graphs of 1000, 3000, 5000, 7000, 10000 nodes from the DMC model, from left to right, with seeds of 5, 8, 10, 20, 50, 100 nodes, from top to bottom.

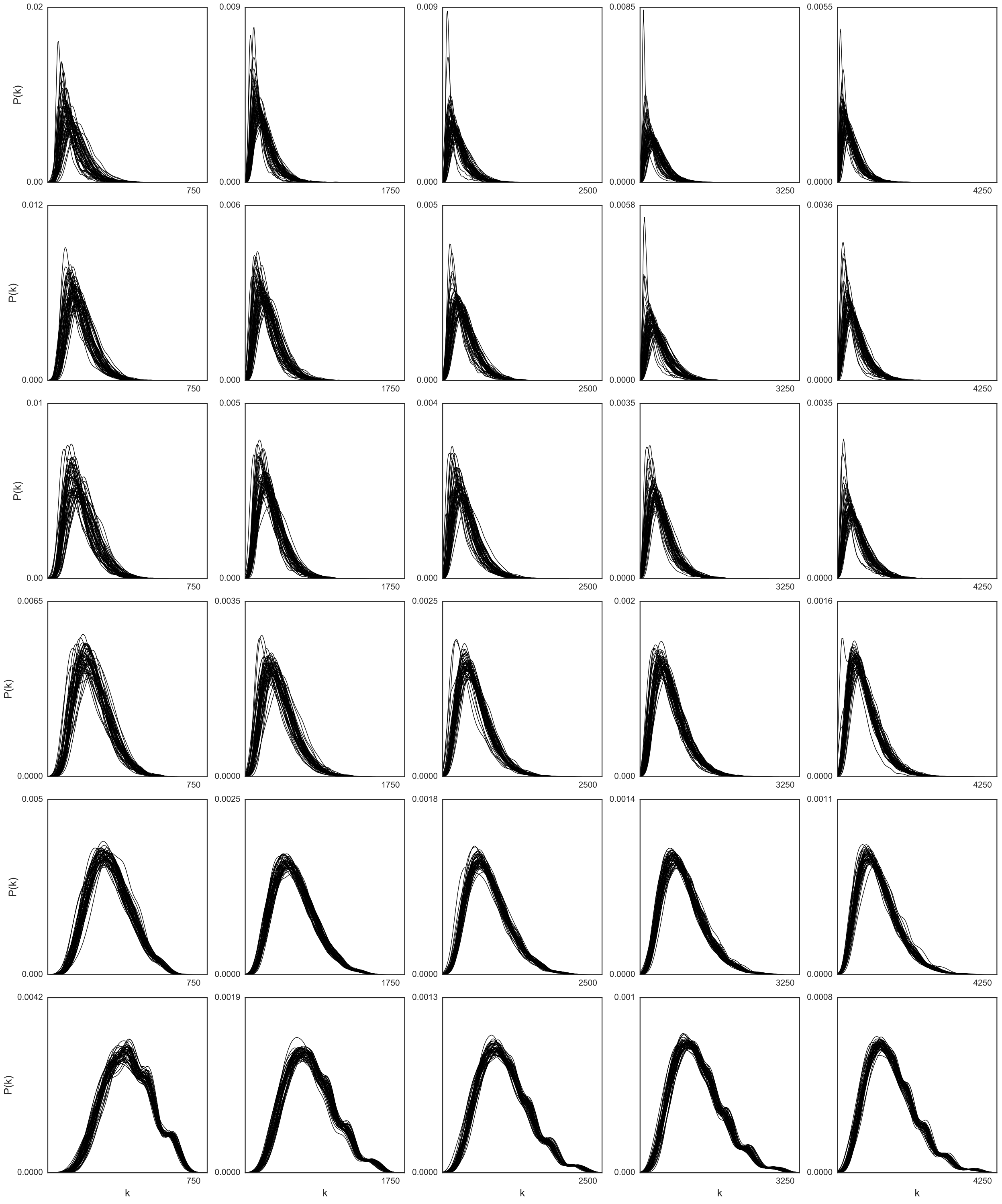

Figure S2: The degree distribution of 50 generated graphs of 1000, 3000, 5000, 7000, 10000 nodes from the DMR model, from left to right, with seeds of 5, 8, 10, 20, 50, 100 nodes, from top to bottom.

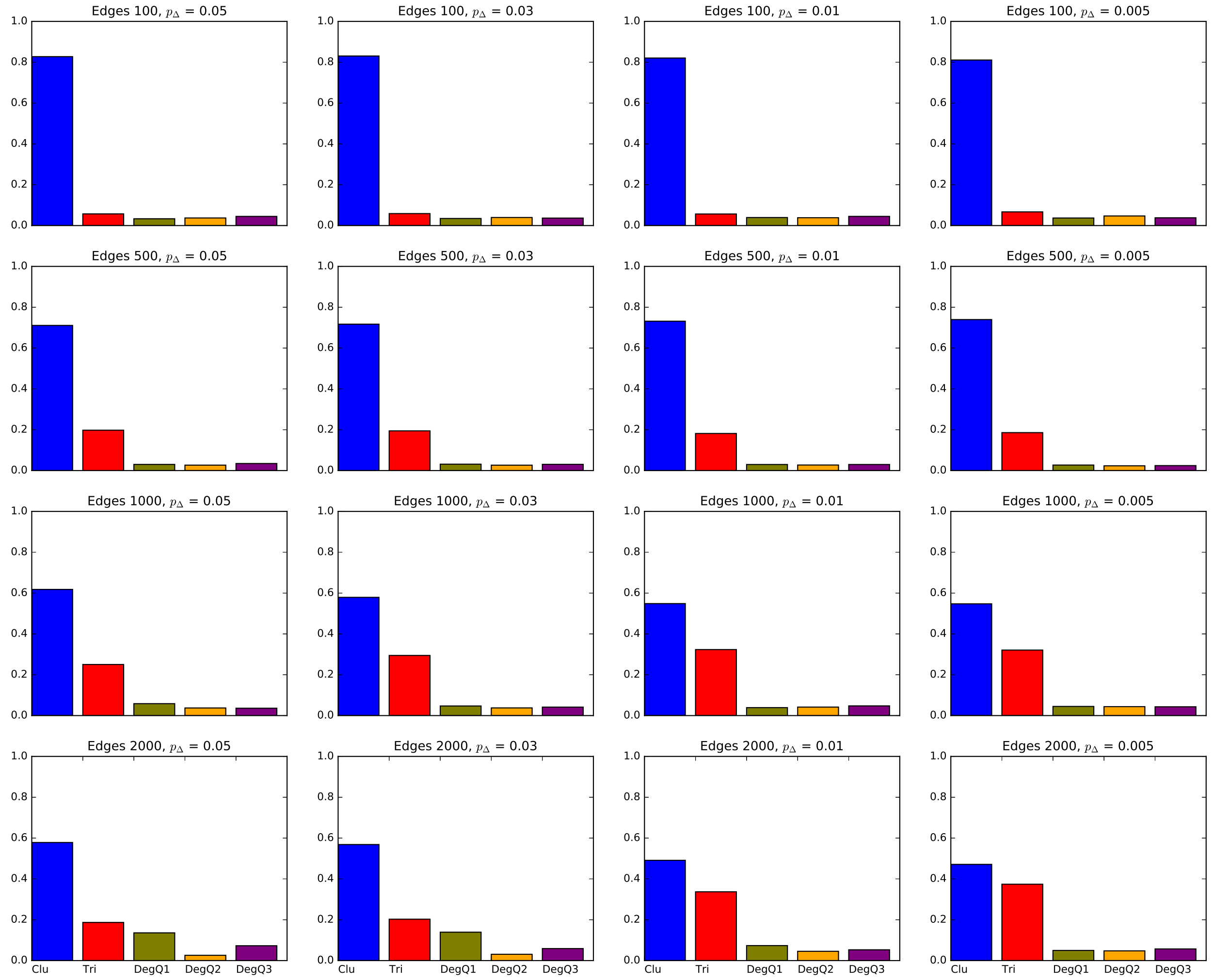

Figure S3: The random forest feature importance in the model selection simulation of the five predictors for p_Δ from 0.05, 0.03, 0.01, 0.005, from left to right, and edge count from 100, 500, 1000, 2000, from top to bottom.

Details for Portion of Nodes to Include in Subsamples

Given the definition of F_1, F_c from the main text, computing the expectation of the KS statistic between the two in general is not possible, since it largely depends on the network model and the seed network used. We will examine this quantity in the setting of the above mentioned $G(n,p)$ variant of the ER model, where the resampling distribution is for the edge count in the induced subgraphs. We chose this model since the induced subgraph of an ER graph is once again an ER graph, so the distribution of the number of edges is still binomial and tractable.

The desired expectation of the KS statistic can then be written as follows, with a few approximations:

$$
\begin{aligned}
& E_G\left[\mathrm{KS}\left(F_1(G), F_c\right)\right] \\
&= \sum_g P(G=g)\,\mathrm{KS}\left(F_1(g), F_c\right) \\
&= \sum_l \sum_{g:|E_g|=l} P(G=g)\,\mathrm{KS}\left(F_1(g), F_c\right) \\
&\approx \sum_l \sum_{g:|E_g|=l} P(G=g)\,\mathrm{KS}\left(\tilde{F}_1(l), F_c\right) \\
&= \sum_l \mathrm{KS}\left(\tilde{F}_1(l), F_c\right) \sum_{g:|E_g|=l} P(G=g) \\
&= \sum_l \mathrm{KS}\left(\tilde{F}_1(l), F_c\right) P\left(|E_G|=l\right)
\end{aligned}
$$

The summation in the first line is over all the possible realizations, indexed by g, of a network G generated by the $G(n,p)$ model. Assuming the proportion of nodes in the subsample is α, then each induced subgraph of a network generated by the $G(n,p)$ model should be $G(\alpha n, p)$, since each of the possible edges of the induced subgraph is still independent and has a probability of p to exist. Thus, F_c is still be binomial $B(C(\alpha n, 2), p)$ and remains constant. On the other hand, F_1 depends on g and is indicated as such. On the second line, a new index l, the number of edges in G, is introduced, with a nested summation for all g such that its edge set E_g has cardinality l. On the third line, we approximate $F_1(g)$ for $\forall g$ such that $|E_g| = l$ with $\tilde{F}_1(l)$, which is a distribution that only depends on l. On the fourth line, we move the term in the inner summation that does not depend on the index of the inner summation into the outer summation. Finally, on the fifth line, we can write the nested summation on the fourth line as $P(|E_G| = l)$, the probability for G to have l edges.

Let $p_l = l/C(n,2)$, then conditional on l, a randomly selected dyad (node pair) from the induced subgraph is an edge with probability p_l. Thus, one reasonable form for $\tilde{F}_1(l)$ would be $B(C(\alpha n, 2), p_l)$. We found this approximation to be accurate only when α is sufficiently small (< 0.3). For larger values of α, this

approximation ignores the increasing effect of correlation between different subsamples due to increasing number of shared dyads, leading to underdispersion when using the $B\left(C\left(\alpha n,2\right),p_l\right)$ approximation. To correct for the correlation, the covariance between two subsamples can be derived exactly, allowing for improved approximation of $\tilde{F_1}\left(l\right)$. Let EC_1 and EC_2 represent the node count from two different subsamples of m nodes from an ER graph of n nodes such that $|E_g|=l$. Then the covariance of EC_1 and EC_2 can be written as:

$$\mathrm{cov}\left(EC_1,EC_2\right)=E\left[EC_1\times EC_2\right]-E\left[EC_1\right]E\left[EC_2\right]$$

The form for $E\left[EC_i\right]=C\left(m,2\right)\times p_l$ is simple, but the first term is more involved. Let $m^*=C\left(m,2\right)$, $o^*=C\left(o,2\right)$, e^i_j be the edge indicator for the jth dyad in the ith subsample, where $\mathbb{O}$ s.t. $|\mathbb{O}|=o$ is the set of nodes that overlap between the two subsamples. Then the second term can be written as:

$$\begin{aligned}
E\left[EC_1\times EC_2\right]&=\sum_o E\left[\left(\sum_{j=1}^{m^*}e^1_j\right)\left(\sum_{j=1}^{m^*}e^2_j\right)\middle|\,|\mathbb{O}|=o\right]\times P\left(|\mathbb{O}|=o\right)\\
&=\sum_o A_o\times B_o\\
A_o&=o^*\times p_l+2\times C\left(o^*,2\right)p_l^2+2\left(m^*-o^*\right)o^*p_l^2+\left(m^*-o^*\right)^2p_l^2\\
B_o&\sim C\left(n,2m-o\right)\times C\left(2m-o,o\right)\times C\left(2m-2o,m-o\right)
\end{aligned}$$

The detailed derivation for A_o and B_o as well as how to generalize the results to other models under dyadic independence and for models without dyadic independence are in the following section.

Two components are required to compute the last line of the expression for $E_G\left[\mathrm{KS}\left(F_1\left(G\right),F_c\right)\right]$. $P\left(|E_G|=l\right)$ is simple to compute since G is $G\left(n,p\right)$, thus $|E_G|$ is distributed according to $B\left(C\left(n,2\right),p\right)$. $\mathrm{KS}\left(\tilde{F_1}\left(l\right),F_c\right)$ is less straightforward, but can be approximated using normal approximations, since the normal approximation for binomial works well for reasonably large "n" ($C(n,2)$ in our case) and values of p not extremely close to 0 or 1. F_c can be approximated with a normal distribution with the corresponding binomial mean and variance, $N\left(C\left(\alpha n,2\right)p,C\left(\alpha n,2\right)p\left(1-p\right)\right)$. For $\tilde{F_1}\left(l\right)$, the naive approximation that ignores correlation is similarly $N\left(C\left(\alpha n,2\right)p_l,C\left(\alpha n,2\right)p_l\left(1-p_l\right)\right)$. However, as stated above, this approximation is inaccurate for larger values of α. We found that an approximation with a normal distribution with mean $C\left(\alpha n,2\right)p_l$ and variance $E\left[EC_1^2\right]-E\left[EC_1\right]E\left[EC_2\right]-\mathrm{cov}\left(EC_1,EC_2\right)$ yields a much closer approximation. Assume that $p\neq p_l$, then it can be easily verified that the maximal difference between the two normal CDFs occurs at x_l, the point where the two normal density functions are equal. Thus, for a particular value of l, $\mathrm{KS}\left(\tilde{F_1}\left(l\right),F_c\right)$ can be approximated by the absolute value of the difference between the two normal CDFs evaluated at x_l.

Next, we examined the relationship between α and $E_G\left[\mathrm{KS}\left(F_1\left(G\right), F_c\right)\right]$ numerically. We computed $E_G\left[\mathrm{KS}\left(F_1\left(G\right), F_c\right)\right]$, with the above approximations, for $n = 1000$, $p = 0.2$, and $\alpha \in \{0.05, 0.1, 0.15, 0.2, 0.25, 0.3, 0.5, 0.6, 0.7, 0.8, 0.9\}$. In addition, we empirically estimated $E_G\left[\mathrm{KS}\left(F_1\left(G\right), F_c\right)\right]$ for each value of α via simulation, where $F_1\left(g\right)$ is estimated from 10000 subsamples of each of 250 independent draws from $G\left(1000, 0.2\right)$ and F_c is estimated from single subsamples of 10000 independent draws from $G\left(1000, 0.2\right)$. The results are summarized in Table S1.

Clearly, $E_G\left[\mathrm{KS}\left(F_1\left(G\right), F_c\right)\right]$ increases with α, although not greatly in the lower range of values of α explored. The naive approximation matches the empirical results closely until about $\alpha = 0.3$, but is very inaccurate for larger values of α. The improved approximation matches the empirical results closely for all values of α and dominates the naive approximation for all values of α examined. The discrepancy between F_1 and F_c does increase with α, but remains small for reasonably small values of α. The improved approximation seems to adhere more closely to empirical results for larger values of α where more nodes are sampled. This is expected since the normal approximation for the binomial distribution improves with larger number of trials. Although this is merely a toy example and the results are by no means general, they do suggest to keep the portion of nodes in the subsample low ($< 30\%$ in this example) as long as enough features of the models can be retained. For instance, taking a single node as the sample would not retain any features and would be too small of a subsample. In addition, this is a cautionary tale about the care needed in choosing the proportion of nodes sampled, since even under dyadic independence, the difference between F_1 and F_c can be noticeably larger than an intuitive approximation for certain values of α.

α	Naive	Improved	Empirical
0.05	0.0158	0.0158	0.0228
0.1	0.0317	0.0319	0.0383
0.15	0.0475	0.0482	0.0518
0.2	0.0633	0.0650	0.0690
0.25	0.0790	0.0821	0.0853
0.3	0.0947	0.0999	0.1033
0.5	0.1559	0.1792	0.1815
0.6	0.1855	0.2265	0.2284
0.7	0.2143	0.2824	0.2835
0.8	0.2422	0.3525	0.3532
0.9	0.2692	0.4517	0.4511

Table S1: Theoretical approximation and empirical estimate of $E_G\left[\mathrm{KS}\left(F_1\left(G\right), F_c\right)\right]$ at various values of α, the proportion of nodes in each subsample. Columns from left to right: naive approximation which ignores correlation, the improved approximation that accounts for correlation, and the empirical estimates.

Additional Mathematical Derivations

Following the uniform nodewise subsampling from the paper for the ER model, to better approximate the resampling distribution, we need to estimate the covariance between different subsamples of the same size from the same ER graph. Say the full ER graph has n nodes, and each subsample contains m nodes. Let EC_1 and EC_2 represent the node counts from two different subsamples of m nodes from an ER graph of n nodes containing $p_l \times C(n, 2)$ edges:

$$\text{cov}(EC_1, EC_2) = E[EC_1 \times EC_2] - E[EC_1]\,E[EC_2]$$

$E[EC_i] = C(m, 2) \times p_l$, so we need to focus on the first term. Let $m^* = C(m, 2)$, $o^* = C(o, 2)$, e_j^i be the edge indicator for the jth dyad in the ith subsample, and $\mathbb{O}$ be the set of nodes that overlap between the two subsamples:

$$E[EC_1 \times EC_2] = \sum_o E\left[\left(\sum_{j=1}^{m^*} e_j^1\right)\left(\sum_{j=1}^{m^*} e_j^2\right)\middle| |\mathbb{O}| = o\right] \times P(|\mathbb{O}| = o) = \sum_o A_o \times B_o$$

We assess the two terms separately, say the first o^* dyads are from the nodes that overlap:

$$\begin{aligned}
A_o &= E\left[\left(\sum_{j=1}^{m^*} e_j^1\right)\left(\sum_{k=1}^{m^*} e_k^2\right)\middle| |\mathbb{O}| = o\right] \\
&= E\left[\left(\sum_{j=1}^{o^*} e_j^1 + \sum_{j=o^*+1}^{m^*} e_j^1\right)\left(\sum_{k=1}^{o^*} e_k^2 + \sum_{k=o^*+1}^{m^*} e_k^2\right)\middle| |\mathbb{O}| = o\right] \\
&= E\left[\left(\sum_{j=1}^{o^*} e_j^1\right)\left(\sum_{k=1}^{o^*} e_k^2\right) + \left(\sum_{j=1}^{o^*} e_j^1\right)\left(\sum_{k=o^*+1}^{m^*} e_k^2\right)\right. \\
&\quad \left. + \left(\sum_{j=o^*+1}^{m^*} e_j^1\right)\left(\sum_{k=1}^{o^*} e_k^2\right) + \left(\sum_{j=o^*+1}^{m^*} e_j^1\right)\left(\sum_{k=o^*+1}^{m^*} e_k^2\right)\middle| |\mathbb{O}| = o\right] \\
&= \sum_{j=1}^{o^*}\sum_{k=1}^{o^*} E\left[e_j^1 e_k^2\right] + \sum_{j=1}^{o^*}\sum_{k=o^*+1}^{m^*} E\left[e_j^1 e_k^2\right] + \sum_{j=o^*+1}^{m^*}\sum_{k=1}^{o^*} E\left[e_j^1 e_k^2\right] + \sum_{j=o^*+1}^{m^*}\sum_{k=o^*+1}^{m^*} E\left[e_j^1 e_k^2\right] \\
&= \sum_{j=1}^{o^*} E\left[\left(e_j^1\right)^2\right] + \sum_{j \neq k \in \{1 \ldots o^*\}} E\left[e_j^1\right] E\left[e_k^2\right] \\
&\quad + \sum_{j=1}^{o^*}\sum_{k=o^*+1}^{m^*} E\left[e_j^1\right] E\left[e_k^2\right] + \sum_{j=o^*+1}^{m^*}\sum_{k=1}^{o^*} E\left[e_j^1\right] E\left[e_k^2\right] + \sum_{j=o^*+1}^{m^*}\sum_{k=o^*+1}^{m^*} E\left[e_j^1\right] E\left[e_k^2\right]
\end{aligned}$$

$$= \sum_{j=1}^{o^*} P\left(e_j^1 = 1\right) + 2 \sum_{j<k \in \{1 \dots o^*\}} P\left(e_j^1 = 1\right) P\left(e_k^2 = 1\right) + \sum_{j=1}^{o^*} \sum_{k=o^*+1}^{m^*} P\left(e_j^1 = 1\right) P\left(e_k^2 = 1\right)$$

$$+ \sum_{j=o^*+1}^{m^*} \sum_{k=1}^{o^*} P\left(e_j^1 = 1\right) P\left(e_k^2 = 1\right) + \sum_{j=o^*+1}^{m^*} \sum_{k=o^*+1}^{m^*} P\left(e_j^1 = 1\right) P\left(e_k^2 = 1\right)$$

$$= o^* \times p_l + 2 \times C\left(o^*, 2\right) p_l^2 + 2\left(m^* - o^*\right) o^* p_l^2 + \left(m^* - o^*\right)^2 p_l^2$$

In the fourth line, the last three terms are all from products of distinct dyads, so the expectation of the product can be separated into product of the expectations. The first term however does contain some products of the same dyad, and needs to be handled differently. For the first term in the fourth line, since the first o^* dyads are the same in the two subsamples:

$$\sum_{j=1}^{o^*} \sum_{k=1}^{o^*} E\left[e_j^1 e_k^2\right] = \sum_{j=k \in \{1 \dots o^*\}} E\left[e_j^1 e_k^2\right] + \sum_{j \neq k \in \{1 \dots o^*\}} E\left[e_j^1\right] E\left[e_k^2\right]$$

$$= \sum_{j=1}^{o^*} E\left[\left(e_j^1\right)^2\right] + \sum_{j \neq k \in \{1 \dots o^*\}} E\left[e_j^1\right] E\left[e_k^2\right]$$

For B_o:

$$B_o = P\left(|\mathbb{O}| = o\right)$$

$$= \frac{\#\text{ of ways to choose two different subsets of } n \text{ elements that have } o \text{ overlapping elements}}{\#\text{ of ways to choose two different subsets of } n \text{ elements}} = \frac{B_o^1}{B_o^2}$$

We need not compute the denominator, but merely normalize the numerator for all possible values of $o \in \{\max(0, 2m-n) \dots m\}$. Note that the union of the two subsets is a set of $2m - o$ elements

$$B_o^1 = (\text{number of ways of choosing } 2m - o \text{ elements out of } n)$$

$$\times (\text{number of ways of choosing the } o \text{ overlapping elements out of } 2m - o)$$

$$\times (\text{number of ways to permute the nonoverlapping } 2m - 2o \text{ elements between the two subsets})$$

$$= C\left(n, 2m - o\right) \times C\left(2m - o, o\right) \times C\left(2m - 2o, m - o\right)$$

These components allow us to compute $\text{cov}\left(EC_1, EC_2\right)$. However, to approximate the variance of EC_i over different subsamples, we will use the expectation of the variance estimator. Say we have taken B

subsamples:

$$
\begin{aligned}
E\left[\hat{\text{var}}\left(EC_i\right)\right] &= E\left[\frac{1}{B}\sum_{i=1}^{B}\left(EC_i-\bar{EC}\right)^2\right] \\
&= E\left[\frac{1}{B}\sum_{i=1}^{B}EC_i^2-\bar{EC}^2\right] \\
&= E\left[EC_i^2\right]-E\left[\bar{EC}^2\right] \\
&= E\left[EC_i^2\right]-\frac{1}{B^2}E\left[\sum_{i=1}^{B}EC_i^2+2\sum_{j<k}EC_jEC_k\right] \\
&= E\left[EC_i^2\right]-\frac{1}{B^2}\sum_{i=1}^{B}E\left[EC_i^2\right]-\frac{2}{B^2}\sum_{j<k}E\left[EC_jEC_k\right] \\
&= \frac{B-1}{B}E\left[EC_i^2\right]-\frac{2}{B^2}C\left(B,2\right)E\left[EC_jEC_k\right] \\
&= \frac{B-1}{B}E\left[EC_i^2\right]-\frac{2}{B^2}\frac{B\left(B-1\right)}{2}E\left[EC_jEC_k\right] \\
&\approx E\left[EC_i^2\right]-E\left[EC_jEC_k\right] \\
&= E\left[EC_i^2\right]-E\left[EC_j\right]E\left[EC_k\right]-\text{cov}\left(EC_j,EC_k\right) \\
&= \text{var}\left(EC_i\right)-\text{cov}\left(EC_j,EC_k\right)
\end{aligned}
$$

The results of using the improved approximation for $E_G\left[\text{KS}\left(F_1\left(G\right),F_c\right)\right]$ that takes the correlation into account as detailed here, as well as the naive approximation and the empirical estimate as detailed in the main text, can be found in **Table** 1 in the main text. There is still some discrepancy even for the improved approximation, but it decreases as the proportion sampled increases. This is likely due to the normal approximation being poor when the number of nodes sampled is small.

Regardless of model, the form of B_o does not change, given uniform random sampling. For models under dyadic independence other than ER, the form of A_o changes due to different moments in terms $E\left[\left(e_j^1\right)^2\right]$ and $E\left[e_j^1\right]E\left[e_k^2\right]$ in the fifth line of the above expression for A_o. For example, with the weighted ER graph as formulated in Garlaschelli (2009), where each dyad is assigned weight W with geometric distribution:

$$P\left(W=w\right)=p^w\left(1-p\right)$$

$$P\left(\text{no edge}\right)=P\left(W=0\right)=1-p$$

$$P\left(\text{edge}\right)=P\left(W>0\right)=p$$

Under this formulation:

$$E\left[\left(e_j^1\right)^2\right] = \frac{p+p^2}{(1-p)^2}$$

$$E\left[e_j^1\right] = E\left[e_k^2\right] = \frac{p}{1-p}$$

For models where dyadic independence does not hold, the $E\left[\left(e_j^1\right)^2\right]$ terms on the fifth line are still the second moment of an individual dyad, but all $E\left[e_j^1\right]E\left[e_k^2\right]$ terms must be replaced with $E\left[e_j^1 e_k^2\right]$ in order to properly account for dependence between dyads. The latter can be obtained from the covariance between dyads as specified by the model.

References

D. Garlaschelli. The weighted random graph model. *New Journal of Physics*, 11(7):073005, 2009.
